# Mucosal Melanoma of the Head and Neck: A Retrospective Review and Current Opinion

**DOI:** 10.3389/fsurg.2020.616174

**Published:** 2021-01-20

**Authors:** Laurence Pincet, Karma Lambercy, Philippe Pasche, Martin Broome, Sofiya Latifyan, Antoine Reinhard

**Affiliations:** ^1^ENT Department, CHUV, Lausanne, Switzerland; ^2^Maxillofacial Department, CHUV, Lausanne, Switzerland; ^3^Oncology Department, CHUV, Lausanne, Switzerland

**Keywords:** melanoma, head and neck neoplasm, late diagnosis, endoscopic surgical procedure, adjuvant radiation therapy, immunotherapy, targeted molecuar therapy

## Abstract

**Introduction:** Head and Neck Mucosal Melanoma (HNMM) is an uncommon malignancy that arises in decreasing order in the nasal cavity, the paranasal sinuses, and the oral cavity. Although radical surgery followed by eventual radiotherapy is acknowledged as the mainstay treatment, patients with advanced stages or multi-focal tumors benefit from new systemic therapies. We wish to share our experience with these treatments and review the current literature.

**Materials and Methods:** We present a case review of every patient treated in our center for an HNMM over the past 10 years, including every patient treated in our center for an HNMM over the past 10 years. We analyzed clinical characteristics, treatment modalities, and outcomes.

**Results:** We included eight patients aged from 62 to 85 years old. We found six MM in the nasal cavity, one in the sphenoidal sinus, and one in the piriform sinus. Six patients underwent endoscopic surgery with negative margins, six underwent radiotherapy with variable modalities. Immunotherapy or targeted therapy was given in cases extensive tumors without the possibility of a surgical treatment or in two patient as an adjuvant treatment after R0 surgery. The three-year overall survival was 50%, and three patients (37.5%) are in remission.

**Conclusions:** HNMM is associated with poor oncologic outcomes regarding the concerned patients of our review, as reported in the literature. New treatments such as immunotherapies or targeted therapies have not significantly changed the prognosis, but they may offer new interesting perspectives.

Our small series of cases seems to confirm that surgical resection with negative margins improves overall survival.

## Key Points:

- Mucosal Melanoma of the Head and Neck is associated with a poor prognosis due to local recurrence and distant metastases.
- Radical surgery remains the cornerstone of management of mucosal melanoma.
- Radiotherapy, as adjuvant or exclusive treatment, seems to provide a benefit on local control and overall survival.
- Immunotherapy gives new perspectives for HNMM's' treatment, either in cases of inoperable tumors or as an adjuvant treatment after surgery.
- Although targeted therapies represent interesting treatment options in cutaneous melanomas, targeted mutations are not similar in mucosal melanoma, and the treatment benefit is less important.

## Introduction

Head and Neck Mucosal Melanoma (HNMM) is a rare type of melanoma, which has increased over the last decades (1). It accounts for 0.03% of all cancer diagnoses and 1 to 4% of all melanomas (1, 2) and involves in decreasing order of frequency the sinonasal cavities, the oral cavity, pharynx, larynx, and upper esophagus (3, 4). No causal risk factor is identified, especially the exposure to UV radiation (1). It is associated with a poor prognosis due to local recurrence and distant metastases. The reported 5-year survival rates vary from 17.1 to 40% (1, 3).

Locally advanced and multi-focal character tumors are the causes of symptoms in HNMM. Extensive surgery may represent a heavy and morbid treatment, involving resection of critical structures with essential functions. The arrival of new systemic therapies, such as targeted or immunotherapy, gives a new perspective. Response rates are, however, lower than those seen in patients with cutaneous melanoma (19 vs. 33%) (5, 6).

It is yet difficult to evaluate the impact of these new treatments, given the rarity of the diagnosis. International guidelines agree to recommend complete resection with negative margins as the first-line treatment and as the best curative option (5, 7). However, they do not express precise positions about the role of radiotherapy and new systemic treatments (8).

We aim to share our experience and present in detail the treatment and evolution of every patient treated in the ENT or oncologic department for a HNMM.

## Materials and Methods

We retrospectively reviewed the records of all patients treated in our hospital for an HNMM over the last 10 years (January 1, 2010 to December 31, 2019). We obtained every living patient's consent to their data treatment and the approval of our institution's legal department to use clinical data for the study case review and to publish de-identified data.

The diagnosis was based on the histopathological analysis of biopsies, and the TNM determined with image reports (head and neck MRI and CT, total body PET-CT).

We used the American Joint Committee on Cancer (AJCC) tumor/node/metastasis (TNM) classification for mucosal melanoma of the head and neck.

We discussed all treatment strategies in the multidisciplinary care team.

We classified adverse effects (AE) according to the Common Terminology Criteria for Adverse Events (CTCAE). It uses a 5-grade scale, from mild to death-related AE.

We analyzed the survival with the Kaplan-Meier method. We used the Kaplan-Meier method to calculate the 1-year and 3-year Overall Survival (OS), Disease-Free Survival (DFS), and Disease-Specific Survival (DSS). We also built the Kaplan-Meier curves for the OS, DFS, and DSS. Likewise, we detailed the survival of T3 and T4 patients and operated or non-operated patients.

OS was calculated between the date the patient was included (diagnosis date) and the study endpoint date or the patient's date of death; Disease-Free Survival (DFS) was calculated between the first surgery and the study endpoint or the first recurrence's date. We used the as surgical date, the latest that gave negative margins on the definitive histological report.

Because we have a small sample of patients, we used the Fisher Test to calculate the *p*-value.

## Results

**Figure 1:**
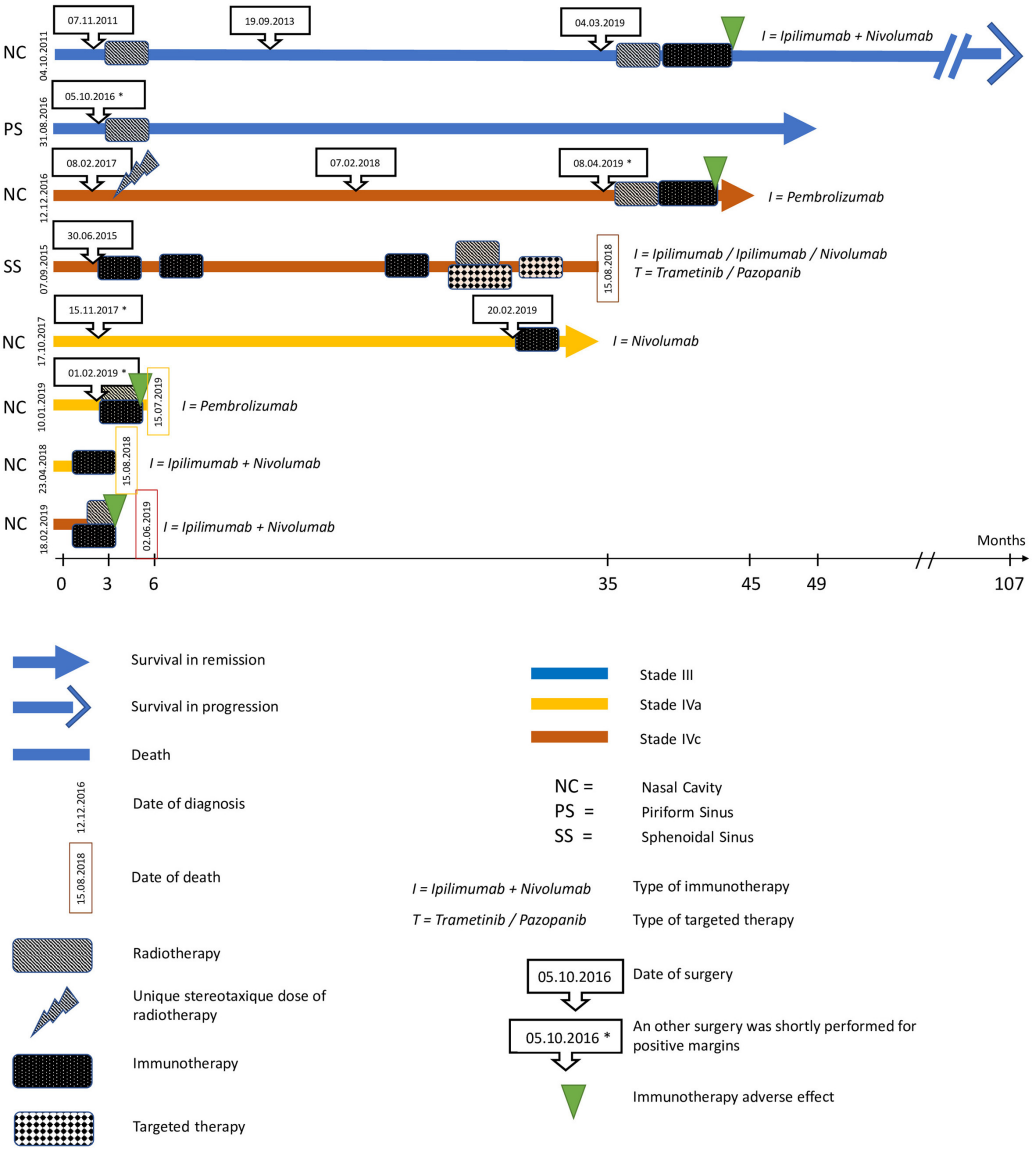
Swimming-plot representing the management of each patient.

We represented every patient's' evolution in a swimming plot (Figure 1).

Eight patients were included, aged from 62 to 85 years old (average: 75y), five women and three men. We identified an exposition to tobacco in the history of three patients. Patients' characteristics are shown in Table 1.

**Table 1:** Patient's characteristics.

Age: median, [range]	75 years [62–85]
Sexe	3 males, 5 females
Ethnie	8 Caucasian
Clinical manifestation:	
- Epistaxis	5 patients
- Nasal obstruction	2 patients
- Chronical cough and dysphonia	1 patient
- Asymptomatic	1 patient
Localization:	
- Nasal cavity	6 patients
- Sphenoïdal sinus	1 patient
- Piriform sinus	1 patient
TNM Classification:	
- T:	
◦ T3	4 patients
◦ T4a	3 patients
◦ T4b	1 patient
- N:	
◦ N0	6 patients
◦ N1	2 patients
- M:	
◦ M0	6 patients
◦ M1	2 patients
Stage:	
- III	2 patients
- IVa	4 patients
- IVb	0 patient
- IVc	2 patients

The most common localization of the HNMM was in the nasal cavity (six patients); one was in the sphenoidal sinus. The main symptom at diagnosis in five of those six patients was an epistaxis and a nasal obstruction in two of them. One HNMM was found in the piriform sinus after multiple examinations for chronic cough and dysphonia. One was found fortuitously in the sphenoidal sinus on a head CT scanner.

Six patients (75%) underwent surgery, among which four were operated on several times for tumor recurrences. We only performed endoscopic surgeries. One patient refused extensive resection with orbital exenteration. The patient with a MM localized in the piriform sinus underwent microlaryngoscopy for the resection.

Anatomopathologists analyzed intraoperative frozen sections and provided definitive analyses within one week. Three patients underwent an early and brief new surgery because of a discordance between the intraoperative frozen sections and the definitive results. All final definitive margins were negative.

We performed a neck dissection for every patient with regional lymph node metastases (N1).

Six patients (75%) underwent radiotherapy, four as an adjuvant treatment after surgery (even with R0 margins), and two as a palliative treatment. We used variables modalities: if 66Gy were usually given (33 sessions of 2Gy), lower doses were administrated in case of proximity with nobles structures such as the optic nerve: two patients received 32.5Gy on the whole tumor (13 fractions of 2.5Gy), and simultaneous boost of 39Gy (13 fractions of 3Gy) distant from the optical nerve. One patient received a unique stereotaxic dose on a brain metastasis.

Seven patients (87.5%) received immunotherapy: monoclonal antibodies against both programmed cell death 1(PD-1) (nivolumab or pembrolizumab) as a monotherapy or in combination with an anti cytotoxic T-lymphocyte antigen 4 (ipilimumab).

Immunotherapy was given as an adjuvant treatment after surgery in two patients, in one patient for an extensive tumor with no possibility of surgical treatment, in three patients for disease progression after surgery and radiotherapy, or as a first-line therapy after one patient refused the surgery.

Immunotherapy caused critical adverse events in four patients. One patient experienced an important asthenia (Grade 3 of adverse event), and another developed a severe myocarditis (Grade 4 of adverse event). For each of them, we had to stop the therapy. Another presented a thyroiditis with severe hypothyroidism (Grade 3 of adverse event) and required a substitutive treatment; the immunotherapy was maintained. One patient died of acute renal failure after an immunotherapy induced severe colitis (Grade 5).

After the failure of three consecutive immunotherapies and a disease progression, one patient received two successive different biological therapies (Trametinib and Pazopanib) with no relevant AE.

**Figure 2:**
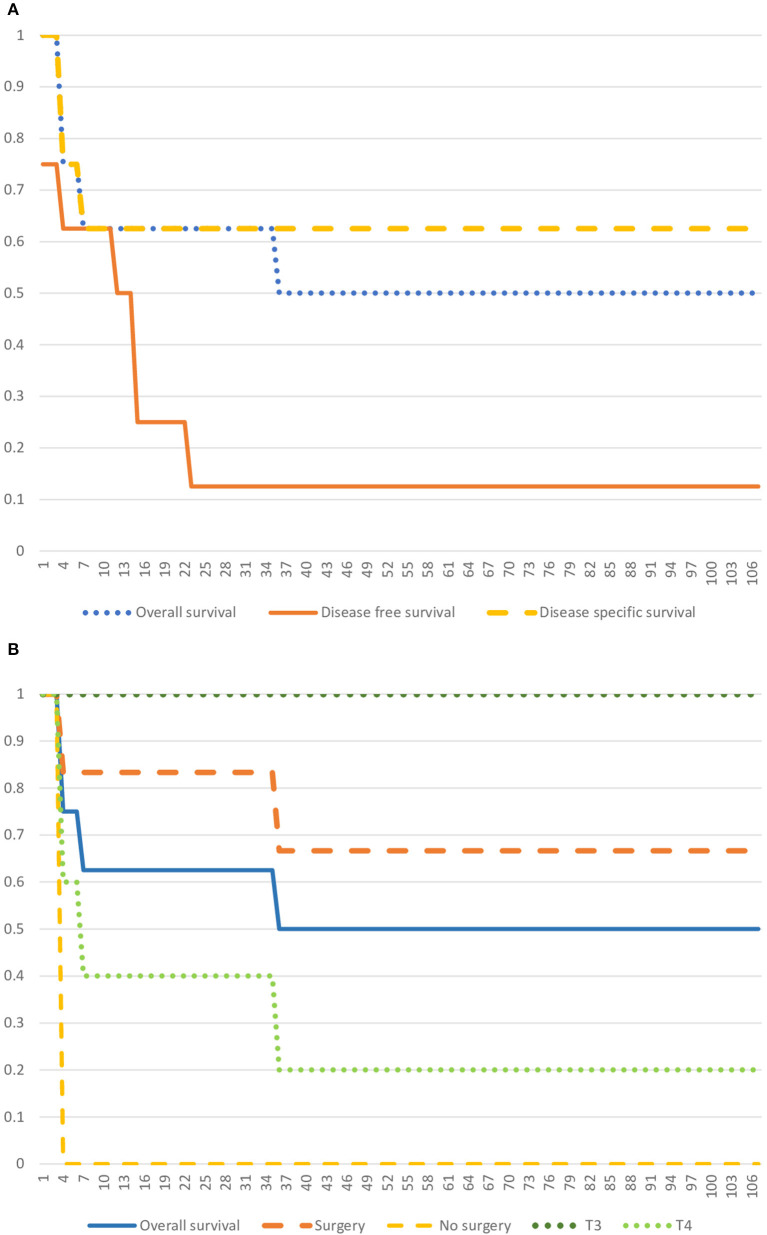
Kaplan Meier Curves. **(A)** Kaplan Meier Curve representing the Overall Survival, the Disease-free Survival, and the Disease-specific Survival. **(B)** Kaplan Meier Curve representing the Overall Survival, and survival specific to T3 and T4 groups, and surgery and no-surgery groups.

The average follow-up was 35.4 months [3-107]. Kaplan Meier curves for OS, DFS, and DSS are represented in Figure 2. One-year OS rate, DFS rate, and DSS rate were, respectively, 62.5; 50; and 62.5%. Three-year OS and DFS rates were, respectively, 50 and 12.5%. Because one patient died from a treatment adverse effect, the 3-year DSS rate remained at 62.5%.

A Fischer-test shows that patients who underwent surgery had a better OS than patients who didn't (*p* = 0.0104). However, there is no significant association between the T stage and OS (T3 vs. T4, *p* = 0.2044). Statistical analysis appears in Table 2.

**Table 2:** Results.

**Global results:**		
	**1-year**	**3-year**
Overall survival rate:	62.5%	50%
Disease-free survival rate	50%	12.5%
Disease-specific survival rate	62.5%	62.5%
Average follow-up (months)	35.4 [3-107]	
Average disease-free survival (months)	27 [16-47]	
**With/without surgery comparison:**	**No surgery**	**With surgery**
Average survival (months)^a^	3.5 [3.4–3.7]	46.6 [6.11–108]
1-year survival	0%	83%
3-year survival	0%	50%
**T3/T4 comparison:**	**T3**	**T4**
Average survival (months)^b^	67.6 [45.6–108]	16.8 [3.4–35.5]
1-year survival	100%	50%
3-year survival	100%	0%

a *A Fisher-test shows a significant difference with p = 0.01*.

b *A Fisher-test shows no significant difference with p = 0.20*.

## Discussion

Our patients' cohort is similar, as reported in the literature. One-year and three-year survival rates (OS, DFS, and DSS) are comparable to other studies. HNMM occurs mostly in patients over 60 years old (2). Sex ratio isn't relevant, even though some authors find a majority of men (4). Epistaxis is the most recurrent initial symptom (2).

As reported in the literature (1, 4), the majority of the MM were diagnosed in the nasal cavity, at advanced stages, without lymph node or metastatic infiltration (N0, M0 stage).

In a large prospective study, including 314 patients, Moya-Plana et al. (8) reported 58.2% in the nasal fossa, 14.5% in the sinuses, and 14.3% in the oral cavity. The majority of the HNMM were T3 (69.3%) and N0 (83.4%). There was no significant correlation between the T stage and the OS.

Due to the rarity of HNMM, there are no established guidelines. Authors agree to recommend to refer patients to a unit with expertise in HNMM and have a multidisciplinary approach (8, 9).

When the tumor was resectable, we favored the surgical approach. Some needed early reoperations because of a discordances between intraoperative frozen sections and final histological reports. In the end, we obtained negative definitive margins for every patient. Statistical analysis showed a significant correlation between surgery and OS. This can reflect that the smaller tumors are more likely to get excised than voluminous tumors. We observed a respectively 100 and 100% survival at 1-year and 3-year in the T3 group. In contrast, it was 50 and 0% in the T4 group. We could not, however, find a significant association between the T stage of the tumor and the OS. Our sample of patients must be too small to get a significant result.

In the current literature, many authors explain HNMM's' high mortality with the advanced stages at the time of the diagnosis. In their opinion, complete surgical resection with negative margins is the best curative option and should be considered as the first-line treatment (1, 3, 5, 7–15).

Australian and USA guidelines recommend surgery for stage III to IVa. On the contrary, for stages IVb or IVc, or when the tumor is close to critical neurovascular structures, surgery gets too destructive or disabling (8).

Absolute obtention of negative margins is still debated: when some small studies found no significant association between positive margins and local recurrence (14), larger studies such as Na'ara's”, show that complete surgical resection with negative margins significantly improves the prognosis of patients with sinonasal MM (9).

In a large prospective analysis of 314 patients, Moya-Plana et al. (1) identified the primary tumor location as a significant risk factor: MM of the nasal fossa is associated with a better prognosis than MM from the paranasal sinus. The authors link this observation with a higher complexity of surgical resection in the paranasal sinus than in the nasal fossa, and therefore the obtention of negative margins.

In a recent update, Lopez et al. (2) reported that failure to achieve local control is correlated with an increased rate of distant disease (from 14 to 71%) and decreased overall survival. Moreover, for patients who fail locally, a new surgery would be capable of salvaging up to 25%.

Difficult interpretation of frozen sections during the surgery is another particularity for mucosal melanoma. Endoscopic tumourectomy consists of piecemeal resections. After tumor resection, the surgeon performs additional circumferential margins to ensure total resection. These margins are then analyzed intraoperatively in a frozen section by pathologists but prove to be difficult without immunostaining. Indeed, in the frozen section, mucosal melanoma may appear melanotic or amelanotic and have various microscopic aspects. The distinction between infiltrated tissue and normal or, even more, inflammatory sinonasal tissue can be extremely challenging. Twenty-five percent of discordance between frozen sections and definitive results is reported (16). That is why immunohistochemistry is so crucial. Pathologists look for a melanoma cocktail antibody [most commonly S-100, human melanoma black (HMB)-45, Melan-A, and microphthalmia transcription factor (MITF)] (16).

Immunohistochemical staining is time-consuming and delays the answer about the surgery's success (R0 resection). Some innovations have been done and propose rapid immunohistochemical staining protocols. However, they are not yet reliable, and surgeons have to wait for the final report. In our experience, we had to repeat four surgeries among the eleven performed (36%) because of a discordance between the frozen sections and the definitive analysis.

Neck dissection is commonly recommended in patients with clinically or radiologically-positive nodes (7, 8). For Moya-Plana et al. (1), cervical node management should be simplified as lymph node metastases have no impact on the prognosis.

Nodal disease is more likely in association with a primary location in the oral cavity than in the sinonasal cavity, both at initial presentation (respectively 25% and 6%) and during the progression of the disease (respectively 42 and 20%) (2, 9). Thus, prophylactic neck dissection could be considered specifically for patients with oral MM.

Sentinel lymph node biopsy can provide an alternative (2). However, radiologists' injection with radiotracer of the primary lesion in the nasal cavity represents an anatomical challenge. For this reason, the sentinel node biopsy is usually not performed in mucosal melanoma.

Melanomas have a poor radiosensitivity, and the use of radiotherapy is controversial. The radiotherapy recommendations and its place in MM's treatment are not clear, and no consensus is reached.

Nevertheless, radiotherapy as adjuvant or exclusive treatment seems to provide a benefit on local control and overall survival.

The Canadian Medical Association and National Comprehensive Cancer Network recommend adjuvant radiotherapy for patients with resected melanoma with high-risk nodal disease (i.e., the presence of four or more lymph metastatic nodes, lymph nodes larger than 3 cm, or macroscopic extranodal soft tissue extension). Patients with positive or close margins who cannot undergo a new surgery also qualify for adjuvant radiotherapy (8). Further clinical trials are warranted to define the role of RT combined with immunotherapy. Studies demonstrate improvement in locoregional control using postoperative radiotherapy. In a large study including 314 patients, Moya-Plana (1) shows no effect of radiotherapy on overall survival (OS) but a clear impact on progression-free survival(PFS) in patients with T3 sinonasal MM. A small case series shows a complete remission in six patients with T3 or T4 sinonasal MM treated with radiotherapy alone (66–72Gy) (17). In the GETTEC study, Benlyazid et al. report a series of 160 patients with localized HNMM treated with surgery alone (*n* = 82) or with postoperative radiotherapy (*n* = 78). The results show a lower locoregional recurrence (29.9 vs. 55.6%; *P* < 0.01) after adjuvant radiotherapy (18). In a similar retrospective study Lai et al. (15) show an improvement of OS (27 vs. 15 months), disease-free survival (DFS) (20 vs.10 months), and 3-year OS (26.9 vs. 15.3%) in favor of adjuvant radiotherapy vs. surgery alone.

The modality of treatment lacks a standardized methodology. A total radiation dose of 60–66Gy is recommended for HN tumors with positive margins after surgery, according to the national comprehensive cancer network (14). Total doses of more than 54Gy and hypofractionation improve local control and overall survival (2, 17). Others showed no superiority of hypofractionated regimens but increased long-term radiotherapeutic complications (2, 3). In some cases, the proximity of the tumor with noble structures (brain stern and optic nerve/chiasma) constraints lower the dose. Those patients don't benefit from the entire effect of radiotherapy.

In cutaneous melanoma, immune checkpoint inhibitors anti-CTLA4 and anti-PD1 and targeted therapies against BRAF and MEK have increased the overall survival. In mucosal melanoma, limited series have shown a lower response rate. Nevertheless, they give new perspectives for the treatment of MM, in particular when facing unresectable tumors, patient's refusal for surgery, or metastatic disease (2, 6, 9, 11).

Ipilimumab (cytotoxic-T-lymphocyte-associated antigen-4-blocker), nivolumab, or pembrolizumab (checkpoint-inhibitor of ligand PD-L1) are the most commonly used molecules. Initially, a combination of adjuvant ipilimumab with nivolumab gave the highest overall response rate vs. each of them in monotherapy (37 vs. respectively 23 and 8%) (1, 9). Recent studies focus on anti-PD1 antibodies since it delivers promising results with an objective response rate of approximately 25–35% and durable clinical response (1).

Ongoing clinical trials for HNMM mainly focus on anti-PD1 immunotherapy with pembrolizumab, associated with radiotherapy after surgery or for unresectable tumors (9). The data shows an improvement in PFS, toxicity, and OS when pembrolizumab is used instead of ipilimumab (9). A durable antitumor activity is also demonstrated regardless of prior treatment with ipilimumab (13).

Clinical trials are ongoing to evaluate the place of neoadjuvant immunotherapy. Moya-Plana et al. (1) suggest neoadjuvant anti-PD1 antibodies as the next step in managing HNMM as the neoadjuvant nivolumab induces significant pathologic response in resectable lung tumors and even in cutaneous melanomas.

A pooled analysis of 86 patients treated with PD-1 blockade alone and 35 patients treated with combined ipilimumab suggested that the combination has a greater efficacy than the monotherapy with a similar safety profile (19) for unresectable or advanced MM. A retrospective study (20) including 35 patients with advanced MM treated with anti-PD-1 produced comparable results of 23% (95% CI: 10–40%) of overall responds rate(ORR), PFS of 3.9 months, and a median overall survival of 12.4 months.

In our case series, we did not significantly improve the patients' survival with immunotherapy. Moreover, immunotherapy was associated with high morbidity and even one death.

Contrary to cutaneous melanoma, the genomic difference might explain MM's lower response rate (21). Patients with a high somatic tumor mutational burden receiving immune checkpoint blockade show an improved survival across a wide variety of cancer, including melanoma (22). Also, mucosal melanoma shows, compared to cutaneous melanomas, a decreased density of tumor-infiltrating lymphocytes. With this less immunogenic characteristic, MM tends to be primarily resistant to immune checkpoint blockade (23).

Another difference with cutaneous melanoma is that the targetable mutations in BRAF and NRAS genes are less prevalent in mucosal melanoma (only 3–15%) (24).

Moreover, most of the BRAF gene mutations affect other regions than codon 600 or are non-activating mutations (5). This hinders the use of BRAF inhibitors. Some MMs are associated with enhanced sensitivity to MEK inhibition. For patients presenting an unresectable tumor or with a mutation on codon 600 (V600E/K), trametinib can give a selective inhibition of MEK1 and MEK2 (24). The combination therapy could delay the development of resistance to BRAF kinase inhibition treatment.

Tyrrell et al. (19) recommend that all mucosal melanoma patients be tested for BRAF mutations given the interesting results obtained for cutaneous melanoma combination of a BRAF and MEK inhibitors treatment. However, no study demonstrates the definite effect of BRAF or MEK inhibitor in mucosal melanoma (25, 26).

On the contrary, aberrations in the KIT gene are more frequent in the sinonasal MM (7–17%) than in cutaneous melanoma and represent a new target (9, 11, 25). Several studies have implicated the efficiency of imatinib in mucosal melanoma (25), and several ongoing trials focus on the effect of KIT inhibition (11). But clinical benefit from targeting this mutated protein appears restricted to specific mutations (25), and errors in the KIT gene are heterogeneous.

Our case series is small and heterogeneous. The arrival of new therapies over the past year regularly changes the landscape of melanomas treatment. Moreover, without guidelines, indication and utilization of those treatments remains dependent on the centre's habits. Thus, interpretation of data is challenging. Literature reviews and meta-analysis should be held to provide more significant results. We aim to share our experience with those new therapies and eventually contribute to further meta-analysis.

## Conclusion

Our small case series agrees with the literature. HNMM is a rare pathology associated with a poor oncologic outcome. New treatments such as immunotherapies and targeted therapies have not yet significantly changed the prognosis of the disease but offer new interesting perspectives for the future.

In agreement with the current literature, our small case series seems to confirm that surgical resection with negative margins improves overall survival.

## Data Availability Statement

The original contributions presented in the study are included in the article/supplementary materials, further inquiries can be directed to the corresponding author/s.

## Ethics Statement

Written informed consent was obtained from the individual(s) for the publication of any potentially identifiable images or data included in this article.

## Author Contributions

LP and AR designed the study. KL, PP, and AR operated the patients. LP wrote the article with the support from KL, PP, and SL. MB contributed to make major revisions in the article following the reviewers recommendations. AR supervised the project. All authors contributed to the article and approved the submitted version.

## Conflict of Interest

The authors declare that the research was conducted in the absence of any commercial or financial relationships that could be construed as a potential conflict of interest.

## References

[1] Moya-Plana A Aupérin A Obongo R Baglin A Ferrand FR Baujat B. Oncologic outcomes, prognostic factor analysis and therapeutic algorithm evaluation of head and neck mucosal melanomas in France. Eur J Cancer. (2019) 123:1–10. 10.1016/j.ejca.2019.09.007 31670075

[2] López F Rodrigo JP Cardesa A Triantafyllou A Devaney KO Mendenhall WM. Update on primary head and neck mucosal melanoma. Head Neck. (2016) 38:147–55. 10.1002/hed.23872 25242350 PMC4986507

[3] Gavriel H McArthur G Sizeland A Henderson M. Review: mucosal melanoma of the head and neck. Melanoma Res. (2011) 21:257–66. 10.1097/CMR.0b013e3283470ffd 21540752

[4] Alves ISS Berriel LGS Alves RT Pinto MB Oliveira CFP Cazzotto AC. Sinonasal melanoma: a case report and literature review. Case Rep Oncol Med. (2017) 2017:8201301. 10.1155/2017/8201301 28255482 PMC5306975

[5] Tyrrell H Payne M. Combatting mucosal melanoma: recent advances and future perspectives. Melanoma Manag. (2018) 5:MMT11. 10.2217/mmt-2018-0003 30459941 PMC6240847

[6] Yentz S Lao CD. Immunotherapy for mucosal melanoma. Ann Transl Med. (2019) 7:S118. 10.21037/atm.2019.05.62 31576325 PMC6685869

[7] Baujat B. Hans S Réseau d'expertise français sur les cancers ORL rares (REFCOR). Oncologie. (2008) 10:363–7. 10.1007/s10269-008-0889-0

[8] Pittaka M Kardamakis D Spyropoulou D. Comparison of international guidelines on mucosal melanoma of the head and neck: a comprehensive review of the role of radiation therapy. In Vivo. (2016) 30:165–70. 10.0000/iv.iiarjournals.org/iv/30/3/165 27107071

[9] Na'Ara' S Mukherjee A Billan S Gil Z. Contemporary multidisciplinary management of sinonasal mucosal melanoma. Onco Targets Ther. (2020) 13:2289–98. 10.2147/OTT.S182580 32214828 PMC7083634

[10] Lee SP Shimizu KT Tran LM Juillard G Calcaterra TC. Mucosal melanoma of the head and neck: the impact of local control on survival. Laryngoscope. (1994) 104:121–6. 10.1288/00005537-199402000-00001 8302112

[11] Spencer KR Mehnert JM. Mucosal melanoma: epidemiology, biology and treatment. Cancer Treat Res. (2016) 167:295–320. 10.1007/978-3-319-22539-5_13 26601869

[12] Manolidis S Donald PJ. Malignant mucosal melanoma of the head and neck: review of the literature and report of 14 patients. Cancer. (1997) 80:1373–86. 10.1002/(sici)1097-0142(19971015)80:8<1373::aid-cncr3>3.0.co;2-g 9338460

[13] Hamid O Robert C Ribas A Hodi FS Walpole E Daud A. Antitumour activity of pembrolizumab in advanced mucosal melanoma: a post-hoc analysis of KEYNOTE-001, 002, 006. Br J Cancer. (2018) 119:670–4. 10.1038/s41416-018-0207-6 30202085 PMC6173747

[14] Caspers CJI Dronkers EAC Monserez D Wieringa MH Baatenburg de Jong RJ Hardillo JAU. Adjuvant radiotherapy in sinonasal mucosal melanoma: a retrospective analysis. Clin Otolaryngol. (2018) 43:617–23. 10.1111/coa.13033 29150980

[15] Lai Y Meng X Liu Q Lu H Guo L Wang S. impact of adjuvant therapy on survival for sinonasal mucosal melanoma. Acta Otolaryngol. (2020) 140:79–84. 10.1080/00016489.2019.1635269 31755795

[16] Chiu AG Ma Y. Accuracy of intraoperative frozen margins for sinonasal malignancies and its implications for endoscopic resection of sinonasal melanomas. Int Forum Allergy Rhinol. (2013) 3:157–60. 10.1002/alr.21075 22972711

[17] Sas-Korczynska B Reinfuss M Mitus JW Pluta E Patla A Walasek T. Radiotherapy alone as a method of treatment for sinonasal mucosal melanoma: a report based on six cases and a review of current opinion. Reports Pract Oncol Radiother. (2018) 23:402–6. 10.1016/j.rpor.2018.07.014 30147451 PMC6105761

[18] Benlyazid A Thariat J Temam S Malard O Florescu C Choussy O. Postoperative radiotherapy in head and neck mucosal melanoma a GETTEC study. Arch Otolaryngol Head Neck Surg. (2010) 136:1219–25. 10.1001/archoto.2010.217 21173371

[19] D'Angelo' SP Larkin J Sosman JA Lebbé C Brady B Neyns B. Efficacy and safety of nivolumab alone or in combination with ipilimumab in patients with mucosal melanoma: a pooled analysis. J Clin Oncol. (2017) 35:226–35. 10.1200/JCO.2016.67.9258 28056206 PMC5559888

[20] Shoushtari AN Munhoz RR Kuk D Ott PA Johnson DB Tsai KK. The efficacy of anti-PD-1 agents in acral and mucosal melanoma. Cancer. (2016) 122:3354–62. 10.1002/cncr.30259 27533633 PMC5134420

[21] Hayward NK Wilmott JS Waddell N Johansson PA Field MA Nones K. Whole-genome landscapes of major melanoma subtypes. Nature. (2017) 545:175–80. 10.1038/nature22071 28467829

[22] Samstein RM Lee CH Shoushtari AN Hellmann MD Shen R Janjigian YY. Tumor mutational load predicts survival after immunotherapy across multiple cancer types. Nat Genet. (2019) 51:202–6. 10.1038/s41588-018-0312-8 30643254 PMC6365097

[23] Kaunitz GJ Cottrell TR Lilo M Muthappan V Esandrio J Berry S. Melanoma subtypes demonstrate distinct PD-L1 expression profiles. Lab Investig. (2017) 97:1063–71. 10.1038/labinvest.2017.64 28737763 PMC5685163

[24] Komatsu-Fujii T Nomura M Otsuka A Ishida Y Doi K Matsumoto S. Response to imatinib in vaginal melanoma with KIT p.Val559Gly mutation previously treated with nivolumab, pembrolizumab and ipilimumab. J Dermatol. (2019) 46:e203–4. 10.1111/1346-8138.14763 30614559

[25] Wang X Si L Guo J. Treatment algorithm of metastatic mucosal melanoma. Chinese Clin Oncol. (2014) 3:1–9. 10.3978/j.issn.2304-3865.2014.08.04 25841464

[26] Tajudeen BA Vorasubin N Sanaiha Y Palma-Diaz MF Suh JD Wang MB. Sinonasal mucosal melanoma: 20-year experience at a tertiary referral center. Int Forum Allergy Rhinol. (2014) 4:592–7. 10.1002/alr.21324 24664639

